# Adapting the EU Economic Governance to New Macroeconomic and Political Realities

**DOI:** 10.1007/s10272-020-0922-0

**Published:** 2020-10-24

**Authors:** Raffaele Fargnoli

**Affiliations:** DG ECFIN on Macroeconomics, European Commission, Rue de la Loi 170/Wetstraat 170, 1049 Brussels, Belgium

## Abstract

There was a widespread consensus on the need to modify fiscal rules in the EU even before the COVID-19 crisis. The aim of this article is to reflect on the reform of fiscal rules from a broader perspective, looking at three different dimensions: the political economy of fiscal rules in the current political and economic environment, the renewed debate about fiscal policy roles and objectives, as well as the current incomplete nature of the European Monetary Union and the prospects for its completion. The main contribution of this paper is to analyse EMU fiscal policy and the related governance modes from a broader perspective. Furthermore, the article briefly discusses ideas for a new model of fiscal and economc surveillance based on a cooperative governance system in order to better fit with with current macroeconomic and political realities.

Even before the COVID-19 crisis, which has led to the suspension of the Stability and Growth Pact (SGP) through the activation of the general escape clause to be invoked in a severe economic downturn, there was a widespread consensus on the need to modify fiscal rules configuration. Current rules are criticised for complexity, procyclicality bias and scarce adaptability to macroeconomic circumstances not driven by very exceptional events. This article is based on Fargnoli (2020) and discusses the reform of fiscal rules across three different dimensions: i) the political economy of fiscal rules in the current political and economic environment; ii) the renewed debate about fiscal policy roles and objectives; iii) the current incomplete nature of the European Monetary Union (EMU) and the prospects for its completion. The reflection over the first two dimensions emphasises that fiscal policy prescriptions stemming from the SGP framework are now suffering from a gap of legitimacy both in terms of ownership (being capable of achieving a synthesis of bold and diverging national preferences) and regarding their output (capacity to deliver on multiple goals). The third dimension highlights the need to rethink the analytical relation between the incomplete nature of the EMU, which is likely to persist in the near future, and the implications for fiscal and economic policy coordination, in particular regarding the need to recover from the coronavirus crisis and to manage its long-term consequences.

* The information and views set out in this article are those of the author and do not necessarily reflect the official opinion of the European Commission.

## The political economy of fiscal rules

The literature on fiscal rules emphasises that their influence on national governments' behaviour stems from (formal enforcement aside) the reputational cost suffered by non-compliant governments. This assumption is based on the ability of external pressure groups – i.e. the public and the market – to offer national policymakers proper incentives (Schuknecht, 2004). In this respect, simpler fiscal rules increase the probability of compliance by facilitating their understanding and monitoring. However, if influential groups fail to exert genuine pressure on national authorities, both simple and better designed rules are just as likely to be neglected as more complex rules. Today, EU fiscal rules have to cope with increasing scepticism about the benefits of strict fiscal discipline as a key principle for sound policymaking. For instance, in some of the countries hardest hit by the pandemic, people have been strongly touched by the often evoked argument pointing to the link between the overwhelmed health care system and previous years' budgetary cuts. In this respect, it is likely that the unprecedented economic fallout triggered by COVID-19, coupled with the political and economic costs inherited from the last financial and sovereign debt crisis, will cement negative feelings towards fiscal consolidation for a long time.

The Great Recession also showed that the market's ability to correctly price the risks related to national economy fundamentals suffers from several flaws. To this extent, the public's confidence in market assessment is also eroding. Thus, sudden increases in sovereign spreads, among other effects, are also likely to cause frustration in relation to the rising demand for balancing fiscal discipline with other policy goals, in particular supporting growth and economic recovery, increasing public investment and reducing unemployment and inequalities. In high-debt countries, because of subdued growth and inflation, the permanent increase in the primary surplus needed to put the evolution of the debt-to-GDP ratio on a convergence path towards the 60% SGP target, ranged, before COVID-19, from 1% of GDP in Belgium and Portugal to 2-3% of GDP in Italy, Spain and France. While the huge stress to which public finances are currently subject will increase these requirements, greater fiscal corrections risk being politically unattainable in the future. Indeed, the social and political backlash, partly triggered by the Great Recession and its policy response, coupled with more recent protests related to relatively modest tax hikes, e.g. by the *gilets jaunes* movement in France, suggest that in a number of countries, the political limits for fiscal consolidation have already been reached.

## The return of fiscal policy

The so-called period of great moderation, characterised by inflation under control, decreasing unemployment and lower output volatility, cemented a widely accepted consensus over fiscal policy functions. Most economists used to agree that fiscal policy should focus mainly on the long-term sustainability of public finances, while leaving stabilisation goals to the mere functioning of automatic stabilisers. This consensus was the basis for the design of the SGP. The limits of monetary policy effectiveness in an environment characterised by interest rates at the zero lower bound hit this conventional wisdom. A general shift in thinking advocates a more active role for discretionary fiscal policy for different purposes such as: i) to cope with present and future stabilisation needs, especially in the presence of hysteresis implying lasting lower output levels (Draghi, 2019); ii) to address deflationary tendencies and the persistent gap in aggregate demand (Rachel and Summers, 2019); iii) to stimulate long-term output through growth-enhancing expenditure (Deleidi and Mazzucato, 2019).

If the persistent 'liquidity trap' started to erode the old consensus, COVID-19 is fully abating it, leading to a profound revolution with regard to the set of macroeconomic tools to be used in this new environment. First of all, there is an almost unanimous agreement on the fact that massive public intervention is needed to cope with the mix of strong supply and demand shocks caused by the pandemic in order to keep the economy afloat. Income support for households, extended unemployment benefits, tax reliefs, government capital injections and guarantees for firms besides health-related costs to contain the pandemic will lead to skyrocketing budget deficits. The difficult challenge of managing the resulting high levels of government debt is also affecting the almost sacred prohibition of monetary financing. Some economists are suggesting radical solutions, such as direct central bank money injections into the economy through so-called helicopter money (Gali, 2020). Without going so far, proposals like perpetual bond issuances with central bank backstops (Tabellini and Giavazzi, 2020) or massive asset purchases undertaken in particular by the Federal Reserve blur the lines between unconventional monetary policy and monetary financing.

Figure 1
**Expenditure composition across EU member states**

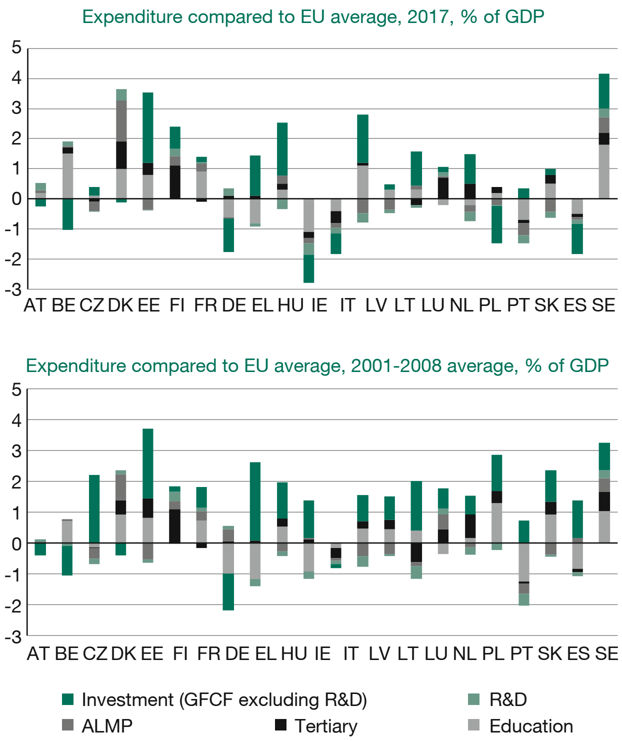

Sources: Eurostat, OECD and author's calculation.

In any case, once the pandemic is finally under control, fiscal policy should be loosened futher to support economic recovery. Before COVID-19, many prominent voices called for profiting from the low interest rate environment to prioritise, over other policy objectives, a powerful investment policy to spur GDP growth both in the short and in the long term (Blanchard, 2019). This debate, reinvigorated proponents of some sort of golden rule to be included in the EU governance framework. However, given the heterogeneity of spending programmes with positive economic returns, ranging from expenditure in education, training, human capital investment, health, climate mitigation, infrastructure and the broad set of innovation policies, it is difficult to conceive an investment clause capable of covering all these items effectively and without creating distortions in the allocation of public expenditure. Figure 1 shows that a number of countries present a suboptimal expenditure composition. Countries with low potential growth rates such as Italy and Spain have expenditures below the EU average in all the growth-enhancing items displayed in the figure, with a

Figure 2
**Institutions, rules and market discipline: The future of the EMU architecture**

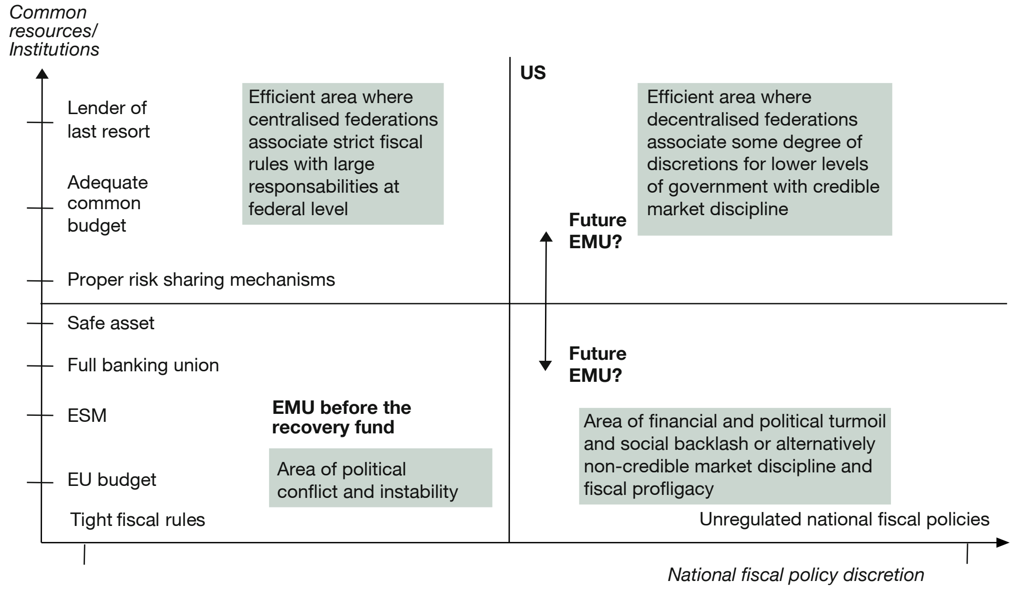

Source: Author's compilation.

gap accounting for almost 2% of GDP. The figure also shows that the expenditure gap widened in recent years, in particular in those countries hit hardest by the sovereign debt crisis.

Decisions on the size and the time extension of counter-cyclical measures as well as the more structural increase in investment will face the limit imposed by debt sustainability concerns. However, since much higher debt levels will become permanent in the future, the debate on how to manage such a high debt burden will intensify. The views will likely diverge between those asking for achieving a larger primary surplus for a long period of time in order to seek reducing the debt ratio and those calling for a strategy aimed at keeping the debt under control in the short term while undertaking well-designed growth-enhancing spending programmes to strengthen the recovery and raise potential growth. In the future passive debt deleveraging based on higher nominal growth and prudent fiscal policy in good times could result in a more substantial and politically sustainable debt reduction (Rawdanowicz, 2013). At the same time, the inherent vulnerability of high-debt countries will raise the question of whether short-term financial market turmoil, causing dangerous increases in sovereign borrowing costs, can be managed by the European Stability Mechanism (ESM) or if, alternatively, either a more active central bank intervention or some form of debt restructuring will become inevitable. Against this backdrop, the debate on fiscal policy choices and debt management interacts with the political economy issues discussed above. Indeed, irrespective of the merit of the different views, this fragile environment will widen the existing divergences about the optimal conduct of fiscal policy, making the current architecture of the European economic governance, in particular the current rules-based fiscal framework, rather unsuitable to the post-pandemic world.

## Fiscal relations and common instruments in an incomplete EMU

Figure 2 displays the main trade-off characterising the prospects for the future evolution in the EMU institutional architecture. On the vertical axes, there are types of common institutions and resources that are typically found in fully fledged monetary unions (federations and confederations). On the horizontal axes, fiscal policy moves from tight fiscal controls exerted by the federal government to larger discretion for lower levels of government. The EMU is now situated in the bottom left quadrant where political tensions and the risk of financial instability are not excluded. This is true despite the creation of the ESM and the fact that the possible completion of the banking union could allow for the reduction of the most extreme consequences of a financial crisis. The EMU, at present, shares some features of centralised models of federations based on the relatively strict fiscal rules often associated with administrative sanctions and mild market discipline since member state bailout is de facto no longer excluded.

However, the EMU lacks the central instruments needed to make this model fully sustainable and resilient. Common schemes to address a common real economy shock are absent. National governments remain reluctant to move towards a minimal fiscal union with stronger spending responsibilities and coercive powers at the EMU level (moving to the upper left quadrant). The resources that will be mobilised at the EU

level through the Next Generation EU instrument could push the EMU (EU) towards the upper quadrants, especially if new EU own budget sources are to be set up and at least part of the common debt is rolled over, to attain a permanent increase in the size of the EU budget. Against this background, it is crucial to highlight a basic principle emerging from present macroeconomic realities and the EMU experience: The larger the common countercyclical and investment instruments permanently available, the stricter the oversight and the constraints on national public finances, also by means of stringent fiscal rules. However, although the agreement on the recovery facility represents a polar step in the right direction by providing crucial support to national budgets, they will continue to play a key role in order to achieve the goals of attaining full recovery and ensuring a proper level of growth-enhancing spending. The temporary nature of the recovery instrument will also require, in the future, that national budgets are able to keep ensuring the financing of some of the main long-term investment programmes activated today by common borrowing. All these considerations support the case for conceiving the future economic governance in a way that guarantees greater room for manoeuvres at the national level, while promoting high quality policymaking and preserving debt sustainability.

Against this background, it has been argued that the peculiar features of the EMU would fit better with a decentralised system based on larger discretion and more cooperation on setting fiscal targets (Wyplosz, 2013). In fact, despite difficulties in the optimal design of fiscal rules and limited transfers flowing from the EU level, full sovereignty on taxation remains a prerogative of member states, who are also likely to retain significant divergences in fiscal policy preferences.

Moving towards a decentralised model would also reinforce market discipline to avoid the risk of fiscal profligacy. These considerations contributed, before COVID-19, to a debate on the opportunity to strengthen the no-bailout rule found in Article 125 of the Treaty on the Functioning of the European Union in order to: i) provide better incentives for sound policymaking and reduce moral hazard risks; ii) scale down the current rules-based system, providing space for tailoring fiscal policy to national preferences within the limits set by market assessments; iii) overcome political resistance towards building up needed common instruments, such as a European Deposit Insurance Scheme.

However, this bright theoretical environment conflicts with reality. Indeed, market assessment is characterised by several flaws, well observed during the Great Recession. Markets' perceptions are pro-cyclically biased as they tend to overlook risks in good times and be inclined to panic in bad times. By overreacting during a crisis period, markets can trigger a vicious circle of continuous increases in interest rates and deterioration of sovereign debt solvency, generating self-fulfilling prophecies, thus turning simple liquidity crises into solvency crises. This can occur more easily in a monetary union due to credit market integration and the free movement of capital across borders which increase the risk of sudden-stop crises, like that experienced by the EMU in 2011-13. Furthermore, the probability of ending in a bad equilibrium scenario will be bigger in the future, as it increases with a larger stock of existing debt.

Indeed, the post-pandemic world, characterised by the strong vulnerability of those EMU countries with a high debt burden, will make the full enforcement of strong market discipline particularly risky. First, the prerequisite to minimise the impact of a sovereign debt default on the real economy and the financial sector have not been created. In the US, the federal budget, besides dealing with stabilisation needs, accounts for key expenditure functions (investment, innovation, top-up of unemployment expenditure, social insurance). This will reduce the real economy cost of an eventual bail-in of a US state, thus increasing the political acceptance of the no-bailout rule and strengthening perceptions about its concrete application (Kirkegaard, 2017). Financial spillovers are also circumscribed by the fact that state government debt is about 13% of GDP and the share of state securities in the portfolio of US banks is low. Despite all of this, the high costs associated with any sort of subnational government default explain why federal governments (in the US and in other federations) often prefer some form of bailout of subnational entities (Cordes et al., 2015).

Furthermore, in the current context, in the EMU, the political impact of debt restructuring risks being even more destructive than its economic costs. The pandemic has amplified the request for recognising mutual interest and mutual support among EU countries. Another reason why a debt restructuring will hardly be accepted is that it will be associated with the last episode of rising public debt, namely the COVID-19 crisis, regarded as an exogenous shock with no government responsibilities involved. Lastly, it should be considered that the downturn induced by a debt restructuring would represent the significant third economic shock in only a few years, after the Great Recession and the current crisis. Against this background, it is likely that strong market discipline will lack credibility in the EMU due to the large economic and political consequences it involves. Alternatively, in case it would entail sovereign debt restructuring, the risk of a political and social backlash triggered by the size of economic disorder that it would generate could jeopardise the EU integration process as a whole.

## Towards a cooperative and robust governance system

These considerations suggest that neither a reform of fiscal rules nor pure fiscal policy decentralisation at the member state level (associated with strong market discipline) are models capable of promoting high-quality fiscal policy and strong political ownership and legitimacy, while also minimising risks of political turmoil and financial instability. In this respect, while these goals could be achieved by building up meaningful and permanent risk-sharing instruments at the EU level, supported by some embryonic form of political union, this scenario does not seem politically attainable for the time being.

An alternative model of fiscal governance could be designed by reducing the role of quantitative fiscal rules and moving towards a cooperative system where fiscal targets and the medium-term macroeconomic strategy are periodically negotiated by a competent EU authority and the national government. The base for negotiations would be represented by a multi-annual plan submitted by the national government at the beginning of its mandate, setting a medium-term debt target and other concrete objectives in terms of fiscal variables and policies. A qualitative analysis of the plan should go hand in hand with the quantitative assessment of government targets. Cooperation and negotiations would serve the purpose of increasing the focus on qualitative variables, e.g. the quality of public expenditure, while also taking into account national views and other policy objectives following an outcome-based assessment. This would increase national authorities' accountability and responsibility, besides providing a high degree of adaptability to macroeconomic circumstances.

This cooperative governance principle could also fit with the underlying governance framework of the Next Generation EU. The narrative and the concrete application of the monitoring system should aim at building up effective cooperation between national governments and EU institutions. For instance, the policy dialogue on the National Reform Programme could serve to: i) identify national policy priorities in need of support; ii) adopt high-quality practices for the design of the spending programmes; iii) monitor implementation and results.

Leaving aside the specific framework underlying the recovery instrument in exchange for more room to manoeuvre with regards to national fiscal policy conduct, a gradual and semi-automatic enforcement system should be put in place to avoid moral hazard. It would range from small penalties in case of simple divergences between EU recommended policies and the national policy strategy, to more serious deterrents for reiteration of ineffective policies and clear-cut gross policy errors. The enforcement tools could include a quasi-automatic contribution to a future stabilisation fund to be regarded as an insurance premium to (partly) cover the risk that the national policy strategy could weaken domestic and EMU resilience.

Finally, in the post-COVID-19 world, a proper management of high levels of public debt will be crucial to safeguard financial stability. In this regard, one could question whether the current Outright Monetary Transactions (OMT) framework would remain fully credible to address sovereign market volatility, in light of the conditionalities attached to the instrument, in particular adhering to an ESM programme (Cohen-Setton and Vallee, 2018). Thus, a broader scope for ECB involvement might be needed in the future. In this respect, avoiding gross policy errors in national budgetary plans could provide the basis for a form of softer conditionality, opening the way to more ordinary ECB intervention, outside a formal OMT programme, to reduce short-term market tensions causing dangerous increases in sovereign borrowing costs.

In general, this cooperative system of governance should be underpinned by an open and inclusive policy dialogue between the EU and national stakeholders. Mutual respect and recognition of a larger range of preferences, views and objectives are the basis for high quality policies to increase input and output legitimacy of EU governance and to underpin trust and support in EU integration. This model could also be used to create an environment where the conditions for moving towards a more efficient fiscal, financial, economic and political union could flourish because it would seed a culture of conflict resolution and would connect national spheres by channelling and mediating different policy conceptions. As John Stuart Mill (1859) asserted in its masterpiece *On Liberty*: "It is only by the collision of adverse opinions that the remainder of the truth has any chance of being supplied" (95).
